# Evaluation of the Wii Balance Board for Walking Aids Prediction: Proof-of-Concept Study in Total Knee Arthroplasty

**DOI:** 10.1371/journal.pone.0117124

**Published:** 2015-01-23

**Authors:** Yong-Hao Pua, Ross A. Clark, Peck-Hoon Ong

**Affiliations:** 1 Department of Physiotherapy, Singapore General Hospital, Singapore, Singapore; 2 School of Exercise Science, Australian Catholic University, Melbourne, Australia; Vanderbilt University, UNITED STATES

## Abstract

**Background and Objectives:**

To provide proof-of-concept for the validity of the Wii Balance Board (WBB) measures to predict the type of walking aids required by inpatients with a recent (≤4days) total knee arthroplasty (TKA).

**Methods:**

A cross-sectional sample of 89 inpatients (mean age, 67.0±8years) with TKA was analyzed. A multivariable proportional odds prediction model was constructed using 8 pre-specified predictors – namely, age, sex, body mass index, knee pain, knee range-of-motion, active knee lag, and WBB-derived standing balance. The type of walking aids prescribed on day 4 post-surgery was the outcome of interest – an ordinal variable with 4 categories (walking stick, narrow- and broad-base quadstick, and walking frame).

**Results:**

Women, increasing body mass index, and poorer standing balance were independently associated with greater odds for requiring walking aids with a larger base-of-support. The concordance-index of the prediction model was 0.74. The model comprising only WBB-derived standing balance had nearly half (44%) the explanatory power of the full model. Adding WBB-derived standing balance to conventional demographic and knee variables resulted in a continuous net reclassification index of 0.60 (95%CI,0.19-1.01), predominantly due to better identification of patients who required walking aids with a large base-of-support (sensitivity gain).

**Conclusions:**

The WBB was able to provide quantitative measures of standing balance which could assist healthcare professionals in prescribing the appropriate type of walking aids for patients. Further investigation is needed to assess whether using the WBB could lead to meaningful changes in clinical outcomes such as falls.

## Introduction

Walking aids such as sticks and frames are recommended for and commonly used by older adults with mobility limitations to promote independent functioning and improve balance.[1,2] Prescribing the appropriate type of walking aids is crucial: inappropriate prescription is by itself a risk factor for falls with often severe consequences.[3,4] Appropriate prescription of the type of walking aids relies, *inter alia*, on an accurate assessment of postural balance.[5] Although the laboratory force-plate is the gold-standard equipment to assess postural balance, healthcare professionals who prescribe walking aids often do not have access to these high-precision but high-cost and non-portable devices. Furthermore, no formal algorithm exists to guide healthcare professionals in the selection of a walking aid.

Recently, we have repurposed the Nintendo Wii Balance Board (WBB) as a portable (3.5kg) balance assessment tool.[6] Whilst we have established the reliability of the WBB-derived center-of-pressure (CoP) measures and their concurrent validity with laboratory force plate measures in healthy and clinical populations,[6,7] it remains untested whether the WBB could have clinical applications such as assisting in the prescription of walking aids.

Therefore, this study sought to provide proof-of-concept for the validity of WBB-measures to predict the type of walking aids required by patients. Noteworthy, we examined inpatients with a recent (≤ 4days) total knee arthroplasty (TKA) – a clinical population with a high risk of injurious falls,[8,9] in which an increase in predictive accuracy would be most clinically important. Furthermore, an acute ward setting was chosen because healthcare professionals have typically no access to a laboratory force-plate in the wards.

## Patients and Methods

### Patients

Our study sample comprised patients aged 50 and older undergoing unilateral TKA for primary knee osteoarthritis at one hospital from June 2010 to January 2011. Patients were recruited within a month before the surgery as part of a randomized trial investigating the effects of postoperative electrical muscle stimulation (ACTRN12610000601033). Patients were excluded if they had previous lower extremity surgery in the past year or were unable to walk 10 meters independently. A total of 104 eligible patients participated in our original clinical study but the present study is concerned with data collected on post operation day (POD) 4 (or earlier on the discharge day). We excluded 15 patients from the final analysis due to the following reasons: (i) underwent unicompartmental knee arthroplasty instead of TKA (*n* = 2); (ii) found to be unfit for the operation (*n* = 6); (iii) declined to continue participation (*n* = 5); and (iv) developed postoperative medical complications that adversely affected the outcomes (*n* = 2). Thus, the final sample comprised the remaining 89 patients. The Singhealth Centralised Institutional Review Board approved this study and all patients provided signed, written, informed consent.

### Candidate independent (predictor) variables

Following a TKA, all patients were managed using a coordinated clinical pathway to ensure standardized medical, pharmacological, and rehabilitation care. Patients included in this study received standard physiotherapy interventions daily from POD1. They also underwent quadriceps muscle stimulation or active quadriceps exercises (control treatment), as per study protocol. For our analysis, beside standing balance (assessed using the WBB), we focused on age, sex, body mass index (BMI), and knee impairments – variables that are routinely or easily obtained in a hospital ward setting and have been correlated elsewhere with activity limitations.[10,11] All data were collected on POD4 by 3 physiotherapists who followed a standardized protocol.

**Knee Pain Intensity**. An 11-point visual numeric pain scale was used to measure knee pain intensity, with 0 indicating ‘no pain’ and 10 indicating ‘worst pain ever experienced’. Two pain ratings – at rest and during leg movement in the past 24 hours – were obtained and a composite average was calculated. The visual numeric pain scale has been shown to be a reliable and valid measure of pain in patients with osteoarthritis.[12]

**Knee Range-of-Motion (ROM)**. A Lafayette Gollehon extendable goniometer was used to measure passive knee flexion and extension ROM. Knee flexion ROM was measured with the patients in supine position. With the assistance of a belt, patients were asked to slide their heels toward the buttocks and to flex their knees maximally. Knee extension ROM was measured with the patients in supine position with the heel elevated on a firm wedge. Two sets of flexion and extension ROM measurements were taken, and the higher ROM measurement was recorded. For the knee ROM measurements, one previous study in patients with knee osteoarthritis has demonstrated good test–retest reliability (intraclass correlation coefficients, 0.95 and 0.85 for flexion and extension ROM, respectively).[13]

**Active Knee Lag**. A Lafayette Gollehon extendable goniometer was used to measure active knee (quadriceps) lag with the patients in supine position with the heel elevated on a firm wedge. Patients were asked perform a straight-leg-raise maneuver which consisted in straightening the operated knee ‘as far as possible’ and lifting the heel ~10cm off the wedge. In this position, knee extension ROM was measured and subtracted by the passive knee extension ROM to represent active knee lag. All patients performed 2 straight-leg-raise maneuvers with a 30-second rest interval, and the smaller active knee lag measurement was recorded.

**Standing Balance**. Standing balance was assessed using the WBB and testing was conducted in the ward gymnasium or in the patient’s cubicle. To perform the test, patients stood unsupported and barefooted on the WBB in their usual comfortable stance and they were instructed to stand quietly. All patients performed two 30-second trials with a one-minute rest interval, and the mean of 2 trials was taken. The WBB was interfaced with a laptop computer using custom-written software (Labview 8.5 National Instruments), and CoP coordinates were recorded at 40 Hz and low-pass filtered at 6.25Hz. Given the myriad of CoP measures that can be extracted from a standing balance test, to avoid spurious (Type I) errors and model overfitting, we *a priori* focused on one measure – the variability or standard deviation (SD) of the CoP around its mean position in the mediolateral axis (ML-SD). Conventionally, higher CoP ML-SD values indicate greater postural sway and hence, poorer balance control. We chose the ML-SD measure primarily because biomechanical studies in older adults showed that walking aids increased ML base-of-support and contributed significantly to frontal (ML) plane standing balance.[14,15]. Furthermore, a recent study in patients with knee osteoarthritis has demonstrated good absolute reliability (standard error of measurement, 9%) and moderate relative (test-retest) reliability (intraclass correlation coefficient, 0.60) for the ML-SD measures [16].

### Outcome Measure

In our study, the outcome measure was the type of walking aids prescribed by one of two principal physiotherapists with a combined experience of over 20 years in orthopedic and sports physiotherapy. Specifically, the type of walking aids was an ordinal variable with 4 categories – namely, walking stick, narrow- and broad-base quadstick, and walking frame (in ascending order of base-of-support provided by the device). To mimic clinical practice, the two physiotherapists were permitted to perform any clinical tests; however, they were masked to the WBB results.

### Statistical Analysis

We used descriptive statistics to characterize the study sample: we used means with SDs for continuous variables and frequencies with percentages for categorical variables. We used the *transcan* function developed by Harrell[17,18] to singly impute missing predictor values in our sample (<3%).

To develop the prediction model for the type of walking aids prescribed – an ordinal outcome with 4 categories – we used a proportional odds regression model which comprised 8 *a priori* predictors listed in Table 1. We confirmed the proportional odds assumption graphically and by logistic regression.[17] As we based our variable selection on prior data/studies, all variables were included in the model without variable selection[19] but we performed a redundancy analysis on the predictors to identify collinear variables. To avoid assuming linearity, continuous measures were modeled as restricted cubic splines unless there was weak evidence against linearity (*P*-value>0.20). Because we used data from a treatment study, treatment assignment variable and its interaction with the predictors were also included in the model. As the Wald joint test for treatment and its interaction terms gave non-significant results, we removed these terms from the final model. Because our sample size was modest, to account for model overfitting, we estimated the odds ratios (ORs) in the multivariable model using penalized maximum likelihood methods.[20] Given that our predictors were measured on different scales, we calculated IQR-ORs to allow valid comparisons between predictors.[17] Specifically, IQR-ORs compared the odds of requiring walking aids with a larger base-of-support between the 75^th^ and 25^th^ percentile levels of the predictors. To facilitate results interpretation, we developed an Excel spreadsheet which gave predicted probabilities for each type of walking aid (S1 Spreadsheet).

**Table 1:** Patient Demographics and Characteristics.

	Walking Aid Type				Overall (n = 89)
	Walking Stick (n = 39)	Narrow-base Quadstick (n = 18)	Broad-base Quadstick (n = 18)	Walking Frame (n = 14)	
Demographics					
Age, mean ± SD	67.3 ± 8.3	65.5 ± 8.1	67.4 ± 6.2	67.6 ± 9.4	67.0 ± 8.0
Female, n (%)	25 (64)	12 (67)	14 (78)	13 (93)	64 (72)
Body mass index, kg/m^2^, mean ± SD	25.8 ± 4.8	27.1 ± 3.8	28.4 ± 5.1	30.0 ± 5.5	27.3 ± 5.0
Knee impairments, mean ± SD					
Knee pain intensity*	2.0 ± 1.6	2.3 ± 1.6	2.8 ± 1.5	2.8 ± 1.6	2.4 ± 1.6
Knee flexion, °	94.4 ± 11.7	92.3 ± 9.3	88.6 ± 7.8	84.9 ± 14.6	91.3 ± 11.5
Knee extension, °	9.0 ± 5.9	12.1 ± 5.4	10.9 ± 5.7	13.4 ± 7.5	10.7 ± 6.2
Active Knee lag, °	4.6 ± 5.2	5.4 ± 3.4	7.9 ± 5.0	7.2 ± 6.6	5.9 ± 5.2
Standing CoP measures, mean ± SD					
CoP ML-SD, cm	0.26 ± 0.09	0.31 ± 0.17	0.34 ± 0.11	0.44 ± 0.20	0.32 ± 0.15

*Assessed using a visual numeric pain scale (0–10), with higher scores indicating worse knee pain.

SD = standard deviation

CoP = center-of-pressure

ML = mediolateral

° degrees

We assessed model performance in 2 ways. First, model discrimination was measured by the concordance index (*c*-index), where a value of 1 represents perfect discrimination and 0.5 represents no discrimination (‘coin flip’). Because a prediction model is expected to perform better (optimistically) in the original derivation sample than in new but similar samples, we used a bootstrap internal validation technique to correct (shrink) the *c*-index for “optimism”.[17] Second, model calibration was assessed using a loess-smoothed calibration plot.

We quantified the predictive performance of CoP ML-SD using the model likelihood ratio *χ* ^2^ statistic and we expressed this value as a fraction of the total likelihood ratio *χ* ^2^ the full model: the larger this fraction, the more contribution from ML-SD to the prognostic information of the full model [17]. To statistically assess the amount of incremental prognostic information contributed by CoP ML-SD, we compared nested models with and without CoP ML-SD using a likelihood ratio test.[17] To further provide a clinical view of its incremental predictive value, we separated patients who were prescribed a walking stick or narrow-base quadstick from those who were prescribed a broad-base quadstick or walking frame, and we calculated the continuous net reclassification improvement (NRI>0) statistic.[21,22] All statistical analyses were done with *R*, version 3.0.1, using the *rms* package.[18]

## Results

Table 1 shows patients’ characteristics. The patients were predominantly female (82%) and were on average moderately overweight. Amongst our 89 patients, 52 (58%) were evaluated as requiring a walking stick; 17 (20%), narrow-base quadstick; 19 (22%), broad-base quadstick; and 19 (22%), walking frame. Table 2 shows the results of multivariable proportional odds model based on 8 *a priori* specified predictors. No redundant variables were identified. Three of 8 predictors were statistically significant: women (*P* = 0.04), those with higher BMI (*P*<0.01) and greater CoP ML-SD (*P*<0.001) were more likely to require a larger walking aid. Knee ROM and active knee lag measures nearly met nominal levels of statistical significance (*P*-values, 0.06 to 0.11).

**Table 2:** Multivariable Association between Predictors and Type of Walking Aids Prescribed.

Variables	Low	High	**Odds Ratio (95% CI)** ^a^	*P*-value
Age	61	74	0.95 (0.44 to 2.06)	.89
Sex	Women	Men	0.32 (0.11 to 0.93)	.04
Body mass index, kg/m^2^	23.4	31.0	3.11 (1.50 to 6.44)	<.01
Knee pain intensity*	1.0	3.5	0.95 (0.44 to 2.08)	.90
Knee flexion, °	85	98	0.60 (0.33 to 1.09)	.09
Knee extension, °	5	15	2.05 (0.95 to 4.27)	.06
Active Knee lag, °	2	9	1.71 (0.89 to 3.28)	.11
CoP ML-SD, cm	0.22	0.41	2.55 (1.23 to 5.30)	<.001

^a^Odds Ratios (ORs) with 95% CIs were derived from proportional odds regression on type of walking aids – an ordinal outcome variable of 4 categories (see further explanation in the text). ORs for requiring walking aids with a larger base-of-support were estimated comparing men with women or the 75^th^ (High) with the 25^th^ (Low) percentile for continuous predictors. For example, other variables being equal, increasing the CoP ML-SD variable from its lower quartile (0.22cm) to its higher quartile (0.41cm) was associated with a 2.55-fold (95%CI, 1.23- to 5.30-fold) increase in the odds of requiring walking aides with a larger base-of-support.

*Assessed using a visual numeric pain scale (0–10), with higher scores indicating worse knee pain.

CoP = center-of-pressure

ML = mediolateral

SD = standard deviation

° degrees

**Figure 1:**
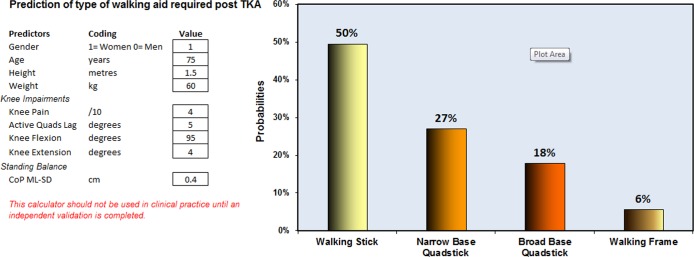
Screenshot of the spreadsheet with calculations of predicted probabilities for each type of walking aid.

**Figure 2:**
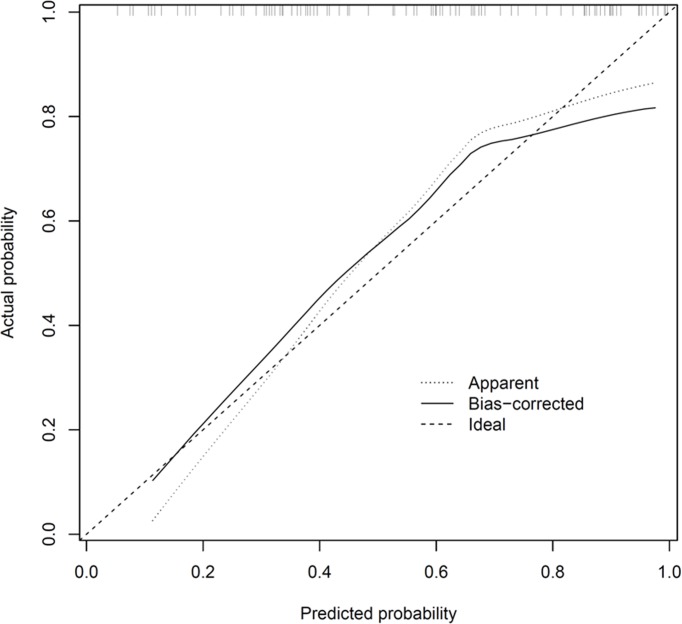
Calibration plot which illustrates the accuracy of the original prediction model (“Apparent”) and the bootstrap model (“Bias-corrected”) in predicting the probability of requiring a quadstick or walking frame. Perfect calibration accuracy is represented by the “ideal” line of unity. Locally weighted scatterplot smoothing is used to model the relationship between observed and predicted probabilities. The distribution of the predicted probabilities is shown as small vertical lines at the top of the graph.

Fig. 1 shows the screenshot of a spreadsheet (provided as S1 Spreadsheet) which allows the user to calculate for individual patients, the predicted probabilities for each type of walking aid. The optimism-corrected *c*-index of the prediction model was 0.74, indicating moderately good discrimination. Fig. 2 shows the calibration plot, with the light dotted and solid lines representing the calibration accuracy of the original and (optimism-corrected) bootstrap models, respectively. The 2 lines were relatively close to the dashed line of unity, indicating moderately good calibration.

**Figure 3:**
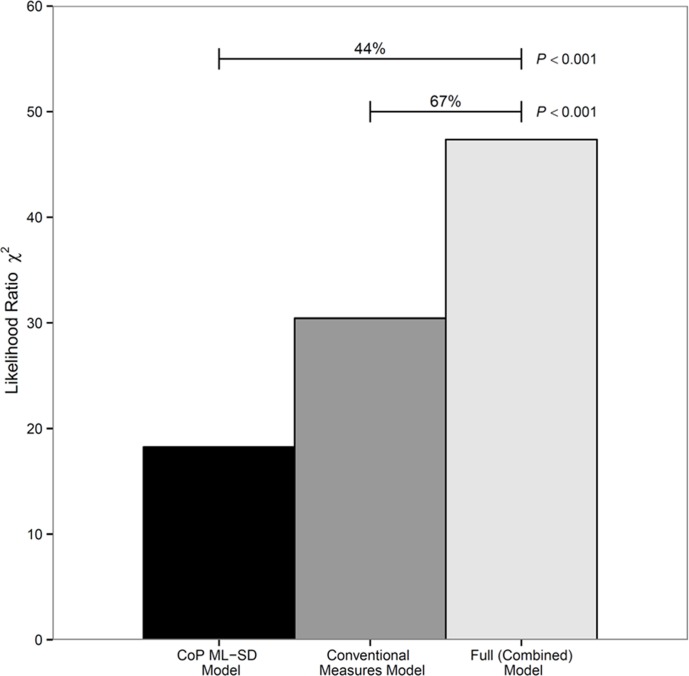
Comparison of predictive value of (i) the full model which comprised CoP ML-SD and conventional measures (demographic and knee variables) and (ii) two nested models which comprised CoP ML-SD or conventional measures. The predictive value is represented by the likelihood ratio *χ* ^2^ statistic. The model comprising CoP ML-SD alone had 44% of the explanatory power of the full model. Put otherwise, nearly half the prognostic information of the full model (comprising conventional and ML-SD variables) may be attributed to ML-SD. Furthermore, ML-SD added statistically significant predictive information (*P*<0.001) to a model that comprised only conventional measures.

Fig. 3 shows the predictive value of models comprising CoP ML-SD or conventional measures (demographic and knee variables), or both. The model comprising only ML-SD had nearly half (44%) the explanatory power of the full model. Put otherwise, 44% of the prognostic information of the full model (comprising demographic, knee, and ML-SD variables) may be attributed to ML-SD. Using the likelihood ratio *χ* ^2^ test for nested models, ML-SD added statistically significant incremental predictive value (*P*<0.001) to a model comprising conventional demographic and knee variables. The NRI>0 associated with the addition of the ML-SD measure was 0.60 (95%CI, 0.19 to 1.01) – a large effect size[23] with a net gain of 38%(12/32; 95%CI, 5 to 70%) in patients who required walking aids with a large base-of-support (broad-base quadstick or walking frame) and 22%(13/57; 95%CI, −2 to 48%) net gain in patients who required walking aids with a small base-of-support (narrow-base quadstick or walking stick).

## Discussion

In our sample of 89 patients with a recent TKA, we developed a model to predict the type of walking aids prescribed for these patients. We found 3 significant predictors: BMI, sex, and standing balance, indexed by CoP ML-SD (Table 2). We also demonstrated that the incremental predictive value of ML-SD beyond conventional predictors was clinically important.

Reviewing the literature, Van der Esch et al[24] reported on 187 older adults with lower limb osteoarthritis and found that functional disability was strongly associated with the possession of walking aids in these patients. Another cross-sectional study by Watson et al[25] on 31 low-care residential older adults showed that the Berg Balance Test – a clinical balance test – was associated with the type of walking aids used. Given that significant or nearly-significant predictors in our prediction model – namely, standing balance, BMI, sex, knee range-of-motion, and knee strength – were correlates of activity limitations in people with TKA,[10,11] our findings corroborated prior studies and provided some confidence for the applicability of the prediction model.

In our prediction model CoP ML-SD was a stronger predictor than conventional knee impairment variables and it had nearly half (44%) the explanatory power of the full model. Importantly, ML-SD more accurately classified patients who required larger base-of-support walking aid to a higher risk group (38% sensitivity gain). Our findings that balance performance was a crucial consideration when prescribing walking aids agree with those by Watson et al.[25] However, their study was based on the Berg Balance Test whereas ours used the WBB. Just 3 or 4 days after a TKA, patients may be unable or unwilling to complete the full battery of 14 Berg Balance Test items and this limitation could potentially create a floor effect. In contrast, all our patients completed two 30-second standing trials on the WBB with no falls or other safety issues, thereby supporting the time-efficiency and feasibility of WBB testing in patients with substantial mobility limitations.

Cost-effectiveness and practicality are additional aspects of the evaluation of new devices. Although the WBB is substantially cheaper than a laboratory force plate, it is not cost-free. Also, healthcare professionals who are facile with movement analysis may feel confident prescribing walking aids without needing information from a WBB. To take a broader perspective, however, the literature identifies a range of healthcare professionals who are involved in prescribing walking aids for older adults, in whom expertise in balance assessment may vary considerably.[26–28] Thus, future studies should evaluate the financial and clinical costs of performing versus not performing WBB testing across different health settings and healthcare professionals. Also of interest, future inter-disciplinary studies could evaluate whether the pedagogical use of the WBB deepens one’s understanding of balance and fall risk assessment.

Our study has limitations. First, our sample size was small which limited our ability to externally validate our model. Accordingly, our prediction model should be viewed as preliminary and requiring further validation and perhaps refinement. Second, although our model showed moderately-good discrimination (*c*-index was 0.74), we acknowledge that a prediction model that incorporates additional variables – for example, fear of falling, home environmental factors, and patients’ preferences – may yield even greater discriminative ability. Third, our findings may not be easily generalizable because we studied only patients with TKA. Future testing is needed to evaluate the added value of WBB information when prescribing walking aids for other high-risk clinical populations. Fourth, we allowed the patients to self-select their stance width, and this may have influenced the results of our study due to the known associations between stance width and CoP variables [29]. However, we specifically chose to test patients in comfortable stance for a number of reasons including ensuring that the position was pain free, easing the implementation and interpretation of the test and enhancing the ecological validity of the findings of the study by replicating a normal activity of daily living.

In conclusion, we provide proof-of-concept that it is possible to obtain objective quantitative measures of standing balance in an inpatient ward setting. More important, our findings suggest that postural sway information from the WBB may help healthcare professionals to more accurately determine standing balance which, in turn, assist in walking aids selection and ultimately prevent falls and related injuries. At a time when existing falls prediction tools have less-than-ideal predictive accuracy in the inpatient setting,[30] our study potentially has important implications for accurate balance assessment in hospitals which contributes to more appropriate prescription of walking aids and prevention of falls.

## Supporting Information

S1 Spreadsheet The spreadsheet used to predict the probabilities for each type of walking aid. (XLS)
